# Interface Mechanics of Double-Twisted Hexagonal Gabion Mesh with Coarse-Grained Filler Based on Pullout Test

**DOI:** 10.3390/ma17010164

**Published:** 2023-12-28

**Authors:** Wenhui Gao, Yuliang Lin, Xin Wang, Tianya Zhou, Chaoxu Zheng

**Affiliations:** 1 School of Civil Engineering, Central South University, Changsha 410075, China; 214807008@csu.edu.cn; 2 Shenzhen Municipal Group Co., Ltd., Shenzhen 518000, China; smec_wx@163.com (X.W.); 13488944472@163.com (T.Z.); 13715999110@139.com (C.Z.)

**Keywords:** pullout test, FLAC3D, interaction mechanisms, gabion mesh

## Abstract

The interface friction mechanics of reinforcement material with filler is an essential issue for the engineering design of reinforced soil structure. The interface friction mechanics is closely associated with the properties of filler and reinforcement material, which subsequently affects the overall stability. In order to investigate the interface mechanism of a double-twisted hexagonal gabion mesh with a coarse-grained filler derived from a weathered red sandstone, a large laboratory pullout test was carried out. The pullout force–displacement curve was obtained by fully mobilizing the gabion mesh to reach the peak shear stress at the interface between the gabion mesh and the coarse-grained filler. The change of force–displacement characteristics and the distribution of tensile stress in gabion mesh during the pullout process were obtained. A 3D numerical model was established based on the pullout test model, and the model for analyzing the interface characteristic between the gabion mesh and the coarse-grained filler was modeled using the FLAC3D 6.0 platform. The interface characteristics were further analyzed in terms of the displacement of soil, the displacement of reinforcement, and the shear stress of soil. The strength and deformation behaviors of the interface during the entire pullout process were well captured. The pullout force–displacement curve experiences a rapid growth stage, a development transition stage and a yielding stabilization stage. The critical displacement corresponding to peak pullout stress increases with the increase in normal stress. The normal stress determines the magnitude of shear stress at the reinforcement and soil interface, and the displacement distribution of a gabion mesh is not significantly affected by normal stress when the applied normal stress is within a range of 7–20 kPa. The findings are beneficial to engineering design and application of a gabion mesh-reinforced soil structure.

## 1. Introduction

The strength of soil can be significantly improved by geosynthetic reinforcement measures. The geosynthetic material is widely used in road foundations, airport, river and coastal engineering [1,2]. Commonly, the geosynthetic materials can be roughly divided into geogrid, geomembrane, gabion, geotextile and geocell. The interface friction mechanism between the soil and the geosynthetic is a key issue for the engineering design of a reinforced earth structure [3,4,5]. The interface friction mechanics is motivated by the friction, occlusion and embedding effect between the soil and the geosynthetic, and subsequently it is influenced by the characteristics of the soil and the geosynthetic, such as the shape of the geosynthetic and the grain composition of the soil [6].

It is difficult to investigate the interface friction mechanics of soil and geosynthetics in the construction field due to the in situ constraints [7,8]. Consequently, the interface friction mechanics of geosynthetics is generally studied by lab test or model test, including a triaxial test, large-scale direct shear test, and pullout test, by which the mechanical behavior of geotechnical structures can be well repeated and investigated [9,10,11,12]. Sidali et al. [13] conducted a series of monotonic consolidated drained and consolidated undrained triaxial tests to study the behavior of reinforced sand. Makkar Femy et al. [14] conducted large-scale direct shear tests to study the interaction between clean dried river sand and a 3D geogrid under direct shear mode. Clemency et al. [15] conducted a series of direct shear tests to investigate the influences of discrete fiber strips on the mechanical properties of reinforced sand and developed a method to strictly control the initial orientation of fiber strips in specimen preparation. Mirzaalimohammadi et al. [16] performed pullout tests on two new systems to reinforce the fine-grained silica, on which transverse steel bars were tied to make a 3D strengthened geogrid. Tang et al. [17] proposed a new wave-shaped fiber as an alternative reinforcement to improve the interfacial mechanical interactions and carried out a series of single-fiber pullout tests to quantify the interfacial shear strength. Moraci et al. [18] performed more than 40 pullout tests on three different HDPE extruded geogrids embedded in a compacted granular soil to analyze the influence of the different parameters on pullout behavior. Among them, the pullout test can elucidate the mechanism of soil geosynthetics, and the results can be directly applied to the specific parameters used in the engineering design of reinforced soil structures. Recently, visualization research including particle image velocimetry [19], the X-ray radiography method [20] and digital image measurement technology [21] has been widely conducted to investigate the microscopic characteristics of geosynthetics and soil during the pullout test.

Numerical simulation is an effective way to investigate the interaction mechanisms of soil and reinforcement. Numerical simulation can well extend the interface friction mechanics of the soil and tensile reinforcement which cannot be observed and measured directly by tests [22,23,24]. Many scholars visualized the load transfer behavior between geogrids and soils in a particle-based view by PFC, providing more insight into the behavior of the interface between geogrids and soils at the microscopic scale [25,26]. Chen et al. [27] developed two types of realistically shaped geogrid models with square and triangle apertures using parallel bonds to reproduce the deformation and strength characteristics of geogrids in PFC3D. Maji et al. [28] and Javdanian [29] developed a numerical model using FLAC to capture the strength and deformation behavior of reinforced sandy. The soil and the reinforcement are analyzed separately by assuming that they interact with each other through friction on the interface. To overcome the above shortcoming, Zhang et al. [30] took the soil skeleton into account while the reinforcing effect was considered as an equivalent additional stress acting on the soil skeleton in the direction of the reinforcement bedding. Gaurav et al. [31] employed FEM-based method to analyze the behavior of a sand square foundation reinforced by geocell. Bhattacharjee et al. [32] established a numerical model of a geosynthetic-reinforced soil-retaining wall with a concrete block by using ANSYS to investigate the horizontal displacement of facing strain developed in backfill soil and reinforcement layers. Sadek et al. [33] adopted PLAXIS to study the advantage of randomly distributed fibers and geogrids in improving the bearing capacity of strip foundations and reducing settlement. Palmeira and Oggeri et al. [34,35] investigated the behavior of reinforced embankments by means of the Distinct Element Method (D.E.M.) and a large-scale cyclic loading test.

In this paper, the interface friction mechanics between a double-twisted hexagonal gabion mesh and a coarse-grained filler was studied by using a large-scale pullout test equipment. Corresponding to the laboratory pullout test model, a numerical simulation was conducted based on the FLAC3D 6.0 platform. The results of the numerical modelling pullout tests were compared with those of the laboratory to further analyze the performance of the displacement and stress of soil and reinforcement and to verify the working mechanism of the reinforcement–soil interface.

## 2. Pullout Test Design

**Figure 1:**
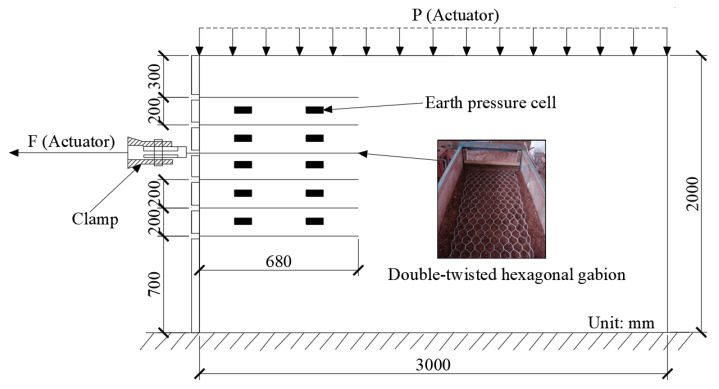
Schematic view of a large-scale pullout test device (reinforcement: double-twisted hexagonal gabion mesh; filler: weathered red sandstone).

The pullout test was conducted in a large model box in Central South University, Changsha, China, as shown in Figure 1. The dimensions of the model box were 3.0 m (length) × 0.85 m (width) × 2.0 m (height). One side of the model box was installed with transparent organic glass to observe the whole pullout test process. The other sides were welded of channel steel and steel plates for sufficient rigidity. To facilitate the pullout test, six layers of a gabion mesh were arranged along the height in the model box, and the spacing between two adjacent layers was 0.2 m. The first layer was 0.3 m from the top of the model box, and the last layer was 0.7 m from the bottom of the model box to reduce the boundary effect. There were two actuators for applying loading. One vertical actuator was fixed at the top of the model box to apply the vertical loading. The other horizontal actuator was adopted to apply the horizontal tensile force through the clamping system at a speed of 0.05 mm/s [36]. A Baylor beam was set at the top of the model box to produce uniform loading. The horizontal actuator could be moved up and down as required. By adjusting the vertical loading provided by the actuator at the top of the model box, the normal stress at the interface between the gabion mesh and the filler could be changed as required. However, due to the influence of frictional resistance on the sides of the model box and the self-weight of the soil, the normal stress on the surface of the gabion mesh could not be well controlled by the vertical actuator. Consequently, it is necessary to set two soil pressure cells in each layer to accurately determine the normal stress on the surface of the gabion mesh.

The coarse-grained filler is a soil derived from weathered red sandstone in the pullout test. The weathered red sandstone is widely distributed in the southern provinces of China. A heavy compaction test was conducted on weathered red sandstone soil. The optimum water content of the weathered red sandstone soil was determined as 18.13%, and the maximum dry density was 1.73 g·cm^−3^. The main physical and mechanical parameters of weathered red sandstone soil are shown in Table 1. Weathered red sandstone can be classified in the sedimentary rock category, ranging from the Tertiary (N) to the Cretaceous (K) period. The mineral composition of weathered red sandstone is predominantly quartz (50%), followed by feldspar (20% to 50%), with the remaining portion consisting of a clay rock fragment and a sandy rock fragment. The particle size of the sedimentary fragment is smaller than 15 mm, indicating a fine to powdery sand-like structure. The main chemical composition of weathered red sandstone mainly consists of nine elements, with the predominant component being SiO_2_, followed by Al_2_O_3_, CaO, and Fe_2_O_3_. The other five chemical components in total make up less than 4%. The particle size distribution of the weathered red sandstone soil used in the pullout test is shown in Table 2.

The gabion mesh is galvanized and covered by a plastic layer. The diameter of the gabion wire is 2.2 mm, and it is 2.7 mm at the edge. The other main technical parameters of the gabion mesh are shown in Table 3. The pullout test model was filled layer by layer by the artificial tamping method, with a thickness of 10 cm for each layer. The controlled compaction degree of weathered red sandstone is approximately 93%, based on the result of a heavy compaction test as the requirement of an actual construction site in Civil Engineering.

**Figure 2:**
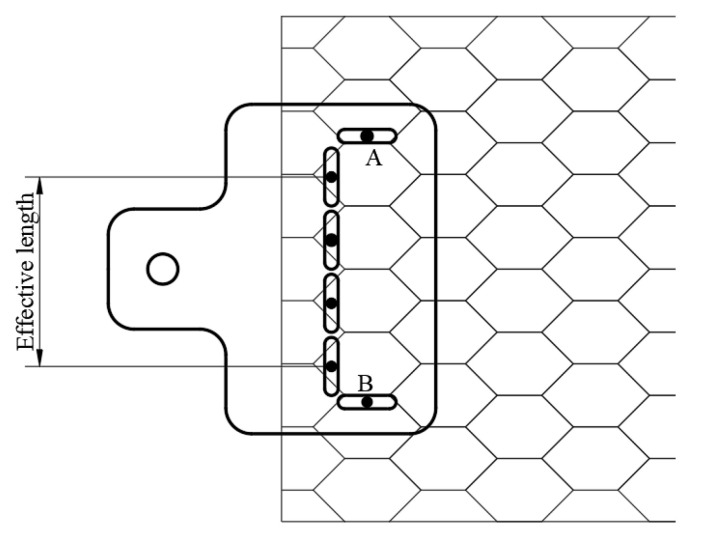
An improved clamping system to reduce the “necking effect”.

The conventional clamping system cannot well fix the hexagonal gabion mesh, and it may produce a “necking phenomenon” during the pulling process and enlarge the measured displacement of the gabion mesh. In order to reduce the “necking effect”, an improved clamping system was developed in the pullout test, as shown in Figure 2. Two identical clamping jigs with two holes (A and B) were used to clamp the gabion mesh, and each hexagonal mesh within the effective length of gabion clamping was fixed tightly with bolts. The lateral constraint at both sides within the effective length can well limit the transverse deformation of gabion mesh and subsequently reduce the “necking effect”.

The pullout displacement and stress of the gabion were automatically recorded by the actuator in the horizontal direction. During the test, the applied normal loading may be partially consumed by the friction resistance along the inner boundary of the model box, which would result in a reduction of the actual normal stress. This phenomenon is related to the size of the model box and specimen. In order to reduce the boundary effect of the model box, a large model box was adopted, and the inner side of the model box was lubricated to decrease the friction resistance. On the other hand, the ratio of specimen width to the box width was controlled.

## 3. Pullout Test Results and Analysis

**Figure 3:**
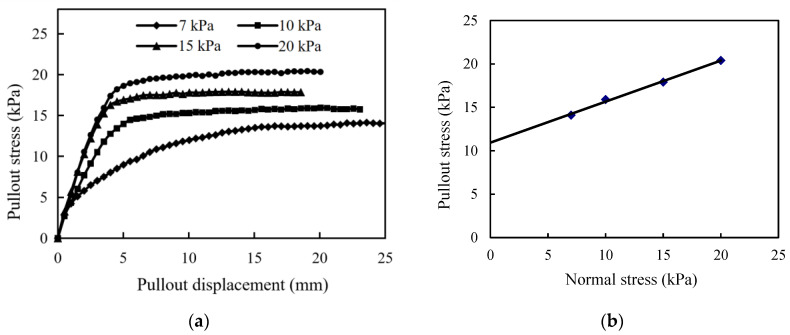
(**a**) Pullout stress versus displacement and (**b**) shear stress versus normal stress in pullout test.

Figure 3 shows the pullout test curves of interface characteristics between the gabion mesh and the coarse-grained filler subjected to different normal stresses in which the pullout stress is equal to the shear stress between the gabion mesh and the coarse-grained filler. The pullout stress gradually increases with the increase in the pullout displacement. When the pullout displacement increases to a certain value, the shear stress does not increase and it finally stabilizes at a certain value. The pullout stress shows the same trend with the increase in the pullout displacement subjected to different normal stresses. The whole curves can be roughly divided into three stages.

(1) Rapid growth stage. When the relative displacement of the gabion mesh and coarse-grained filler is small, the reinforcing effect of the gabion mesh is not fully developed yet, and the pullout stress increases linearly with the increase in the pullout displacement. In this stage, the ratio of shear stress to pullout displacement is almost constant. Since the pullout resistance (consisting of three parts: biting force, friction force and adhesion force) has not been fully developed, it increases linearly with the increase in displacement.
(2) Developing transition stage. The shear pullout increases non-linearly as the pullout displacement continues to increase. The increasing ratio of pullout stress to displacement gradually decreases. It is more difficult to pull out the reinforcements buried deeper in the coarse-grained filler due to a greater friction.
(3) Yield stabilization stage. When the pullout displacement of the reinforcement reaches a critical value, the shear stress remains constant. In this stage, the shear stress does not increase at all when the pullout displacement increases.

The interface frictional parameters of gabion mesh are obtained based on the pullout test, as shown in Table 4. The interface friction angle is 25.3°, and the pullout coefficient is 1.23. There is a mutual extrusion between the hexagonal mesh of the gabion and the surrounding soil under the normal stress, which increases the interface friction, and the occlusion force is generated by the interlocking action between soil particles.

## 4. Numerical Modeling of Pullout Model

**Figure 4:**
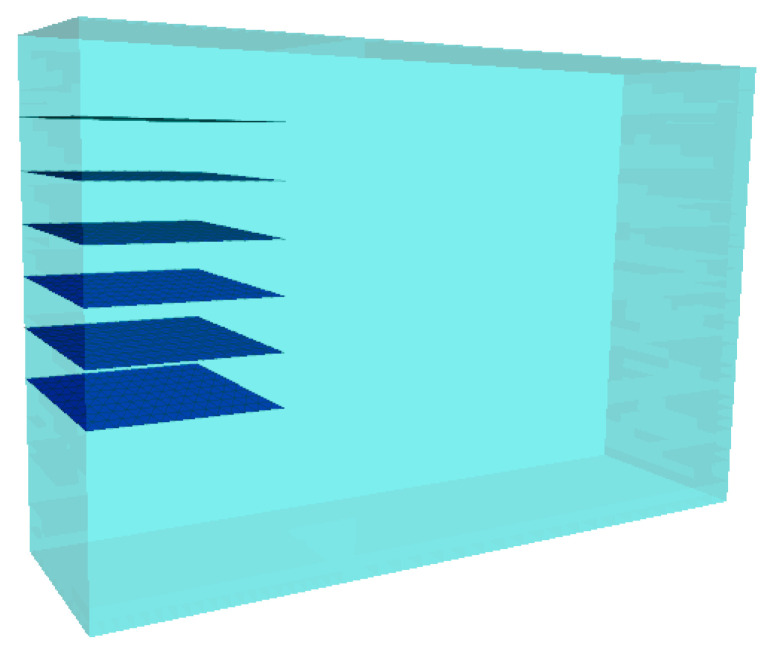
Numerical modeling of pullout test of hexagonal gabion mesh reinforcement.

The numerical model of reinforcement and filler can be established to extend the interface characteristic of the gabion mesh and the filler. Based on the above-mentioned pullout test model, a 1:1 numerical model of the gabion mesh and the filler was established based on FLAC3D 6.0 platform. The size of the numerical model is 3 m × 0.85 m × 2 m, as shown in Figure 4. Corresponding to the laboratory pullout test, the spacing of reinforcing material is 0.2 m, and no reinforcing material is set at a depth range of 0–0.3 m from the top of the model, and no reinforcing material exists within a depth range of 0–0.7 m from the bottom of the model either.

### 4.1. Material Modeling

The soil was modeled by using a zone element, which assumes that the filler obeys the Mohr–Coulomb yield criterion. The twisted hexagonal metal gabion mesh was simulated by a geogrid structural element in FLAC3D 6.0. Based on the results of geotechnical and laboratory pullout tests, the interface friction angle is 25.3°, and the pullout coefficient is determined as 1.23. The remaining parameters are consistent with those used in the laboratory pullout test.

### 4.2. Boundary Condition

The boundary conditions are consistent with the actual conditions in the laboratory pullout test [37]. The *x*-, *y*-, and *z*-directional displacements were continuously constrained, and they were set as 0 at the bottom of the model. The *x*-directional constraints were applied to the left and right sides of the model, and the *y*-directional constraints were applied to the front and rear edges. No constraints were applied to the inner and upper surfaces.

### 4.3. Pullout Process

A strain-controlled pullout simulation was performed with a constant horizontal velocity of 0.05 mm/s at the geogrid node. The normal stress on the interface of the gabion mesh can be changed by adjusting the vertical load applied at the top of the model. Corresponding to the pullout test, the normal stress is set at 7 kPa, 10 kPa, 15 kPa and 20 kPa, respectively. The pullout displacement, the displacement and shear stress of reinforcement, as well as the horizontal displacement and shear stress of the filler, were monitored during the whole pullout process.

## 5. Numerical Results and Discussion

### 5.1. Pullout Curves

**Figure 5:**
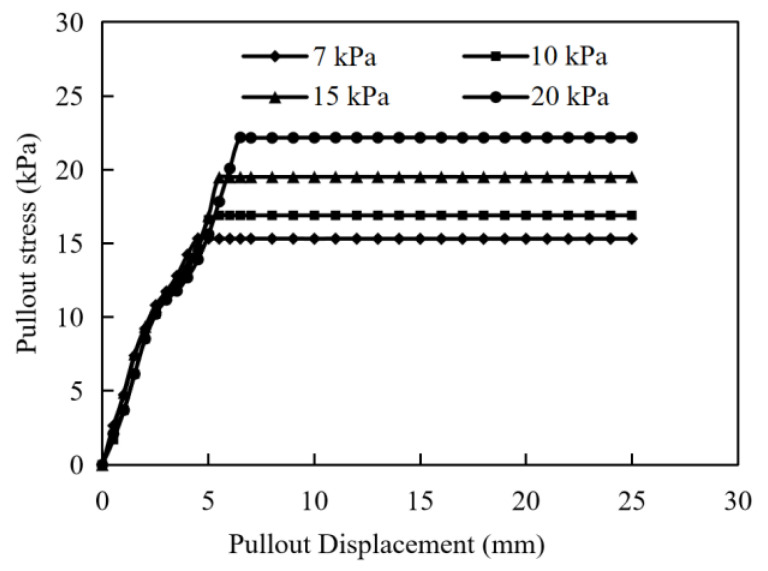
Pullout curves of numerical model.

The pullout curves of the gabion mesh with a coarse-grained filler are shown in Figure 5. It is considered that the numerical simulation result is in a good agreement with that of the laboratory pullout test. The pullout stress gradually increases with the increase in displacement, and it remains constant after pullout displacement reaches a specific value. However, the pullout curve of the numerical simulation also exhibits the following different characteristics compared with those of the laboratory test.

(1) Under different normal stresses, the trend of pullout stress versus displacement determined by numerical simulation is exactly the same at the early stage. The critical displacement corresponding to peak pullout stress increases with the increase in normal stress. This is not exactly the same as the results of laboratory tests. Only when the normal stress is large (larger than 15 kPa) does the pullout stress show the same trend with the increase in pullout displacement at the early stage. When the normal stress is small (less than 15 kPa), the increasing rate of pullout stress versus displacement increases with the normal stress. In laboratory tests, the pullout process is carried out more easily with a lesser normal stress, resulting in the pullout stress lagging behind the displacement.
(2) The pullout test curves obtained by numerical simulation have a more obvious turning point from the rapid growth stage to the yield stabilization stage, while the pullout test presents a relatively gentle development transition stage. The pullout performance of the reinforcement in the laboratory test is achieved through the embedding, friction and occlusion of the hexagonal wire-stranded mesh with the filler, and this effect is gradually exerted with the increase in the pullout displacement. In contrast, the reinforcing effect of the numerical model is calculated by applying parameters such as friction and the cohesion of the coupling spring to the structural geogrid element.

**Figure 6:**
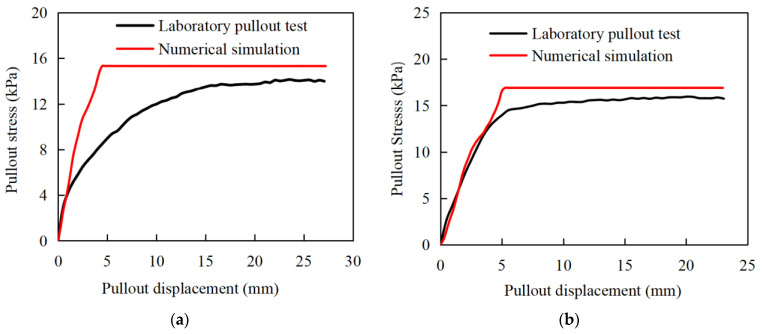

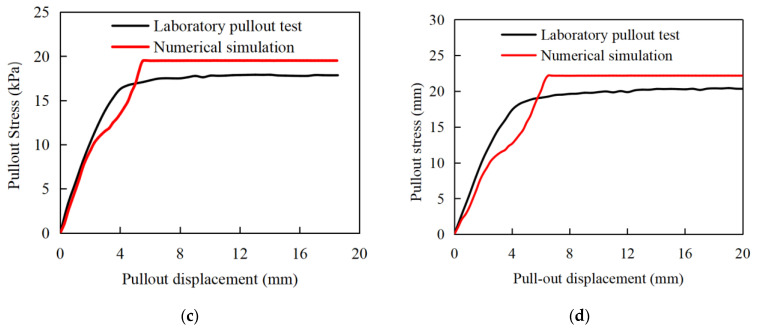
Pullout curves of laboratory pullout test and numerical simulation under different normal stresses. (**a**) 7 kPa; (**b**) 10 kPa; (**c**) 15 kPa; (**d**) 20 kPa.

Figure 6 compares the pullout curves of laboratory tests and numerical simulation under different normal stresses. The peak pullout stresses derived from numerical simulation are larger than those from laboratory tests under different normal stresses. The critical displacements corresponding to the peak critical pullout stress in numerical simulation are also slightly larger than those determined by laboratory tests. As the normal stress increases, the critical displacements in laboratory tests become more and more obvious, and the agreements with the numerical simulation increase. In addition, it is found that the rapid growth phase of the pullout curve in the numerical simulation is not so smooth as that in laboratory tests, which is related to the pullout rate. In the yield stabilization phase, the pullout curves obtained from numerical simulations are nearly stable, but the laboratory test results are not so stable. In the yield stabilization phases of pullout curves, the pullout stress increases at a very small rate with the growth of the pullout displacement. It indicates that the gabions still have a residual pullout resistance, which is gradually consumed during the pullout process.

The peak pullout stress between the pullout test and the numerical simulation of a double-twisted hexagonal metal gabion are compared, as shown in Table 5. The peak pullout stress determined by numerical simulation is generally consistent with that obtained by the pullout test, although the numerical simulation slightly overestimates the peak pullout stress; it is entirely acceptable in engineering practice, with a maximum error of just 11.27% between the pullout test and the numerical simulation. Since the geogrid structural element is adopted to simulate the gabion mesh in the numerical simulation, the double-twisted hexagonal shape of the gabion mesh cannot be well reflected by the numerical simulation, which perhaps is the main cause of such a difference.

### 5.2. The Displacement of Reinforcement

**Figure 7:**
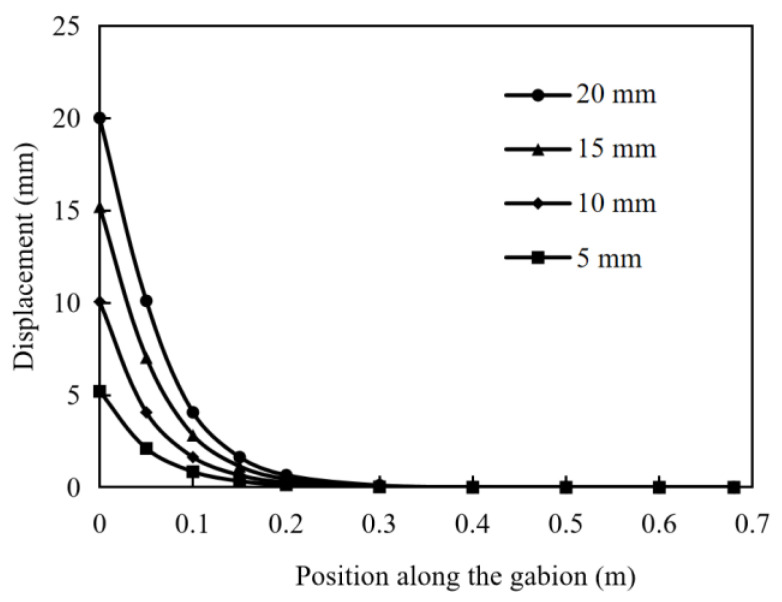
Distribution of displacement along the gabion at pullout displacements of 5, 10, 15 and 20 mm (normal stress: 7 kPa).

In the numerical simulation with a normal stress of 7 kPa, the distribution of the pullout displacement along the length of the gabion is shown in Figure 7. The displacement of the gabion decreases non-linearly along the length, which indicates that the decay of the pulling force mainly occurs in the outer part. As the pullout displacement increases, the displacement reduction rate of the gabion from the clamp to the interior increases, indicating that the pullout force attenuates more. The performance of gabion mesh is fully demonstrated near the clamp position of 0–0.2 m.

**Figure 8:**
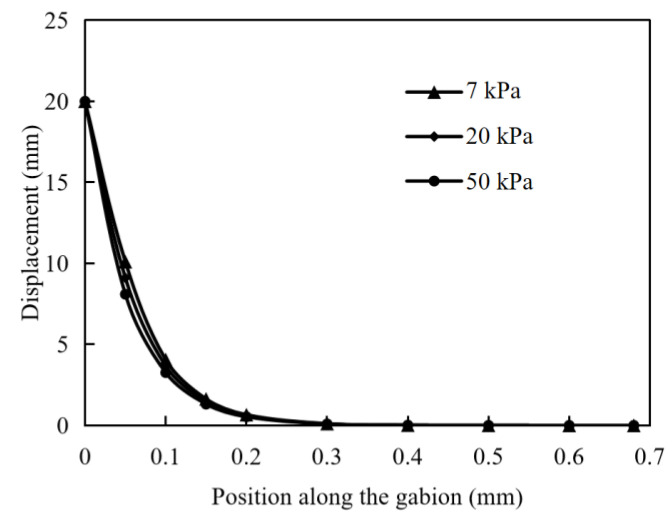
Distribution of displacement curve along the gabion at normal stresses of 7, 20, and 50 kPa (pullout displacement: 20 mm).

In the numerical simulation with normal stresses of 7, 20 and 50 kPa, the distribution of displacement curves along the gabion is shown in Figure 8. Under different normal stresses, the displacement distribution versus length of reinforcement is exactly the same. As the pullout displacement in the numerical simulation increases, the normal stress only determines the magnitude of the shear stress at the reinforcement and soil interface and does not affect the displacement distribution of the reinforcing material. It is considered that the normal stress only affects the difficulty of pulling the reinforcement out of the soil and does not affect the behavior of the reinforcement against the pulling force.

### 5.3. The Horizontal Displacement of Soil

**Figure 9:**
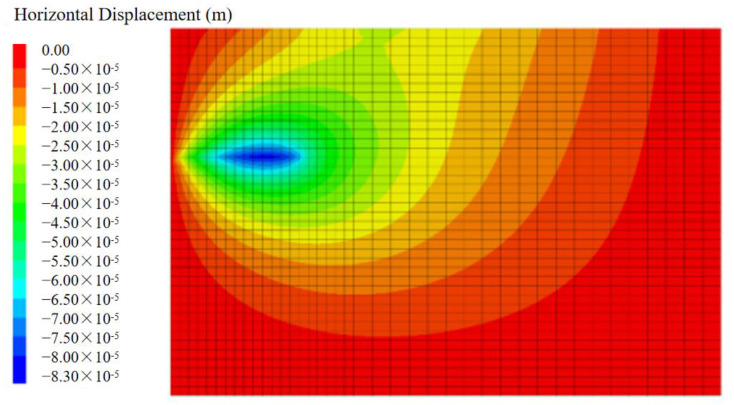
The horizontal displacement distribution of soil.

In the numerical simulation of pullout tests with a normal stress of 7 kPa, the horizontal displacement distribution of the soil at the end of the process is shown in Figure 9. Because of the interfacial friction between the reinforcement and the soil, the reinforcement drives the surrounding soil to move in the direction of the pullout displacement. However, the front side of the model box plays a limited role in the movement of the soil, and the displacement of the soil here is very small. As a result, the horizontal displacement distribution of the soil shows a “ripple” shape, centered on the rear half of the reinforcement and decreasing to the surrounding area.

**Figure 10:**
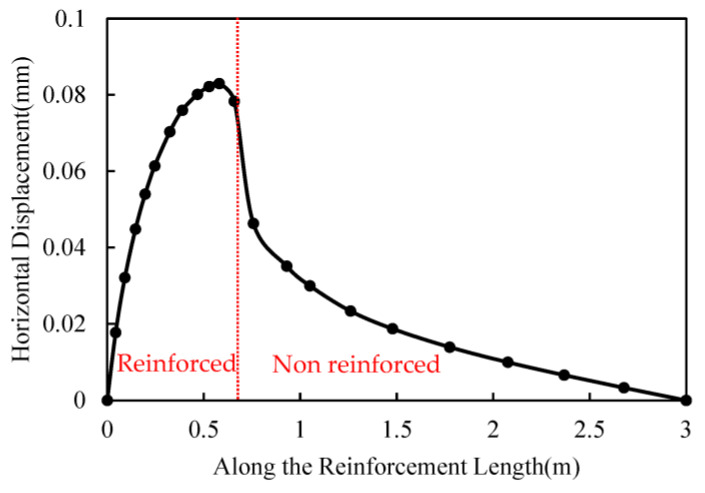
The horizontal displacement of the soil along the length direction of the reinforcement.

The horizontal displacement distribution of the soil along the length direction of the reinforcement in the plane of the pullout process is shown in Figure 10. The horizontal displacement distribution of the soil is in a “bell” shape along the length direction of the reinforcement. Under the action of interface friction between the reinforcement and the soil, the horizontal displacement of the soil gradually increases from the outside to the inside in the reinforced area. In the unreinforced area, the displacement of the soil gradually decreases from its maximum value to 0 in a slow trend. It is worth noting that the maximum horizontal displacement of the soil occurs near the end of the reinforcement soil contact surface, but not completely.

**Figure 11:**
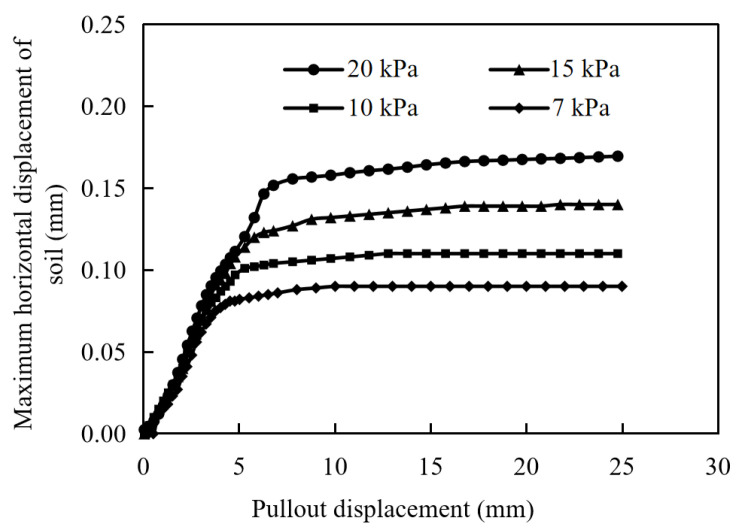
The horizontal displacement distribution of soil versus pullout displacements.

The peak node of the horizontal displacement of the soil is monitored, and the maximum horizontal displacement of the soil versus the pullout displacement under different normal stresses is shown in Figure 11. The shape of the horizontal displacement distribution remains constant with the increase in the pullout displacement, while the values are slightly different. Corresponding to Figure 5, the horizontal displacement of the soil increases with the increase in the pullout displacement in the rapid growth stage and keeps stable in the yield stabilization stage. As the normal stress increases, the maximum horizontal displacement of the soil also increases.

### 5.4. The Shear Stress of Soil

**Figure 12:**
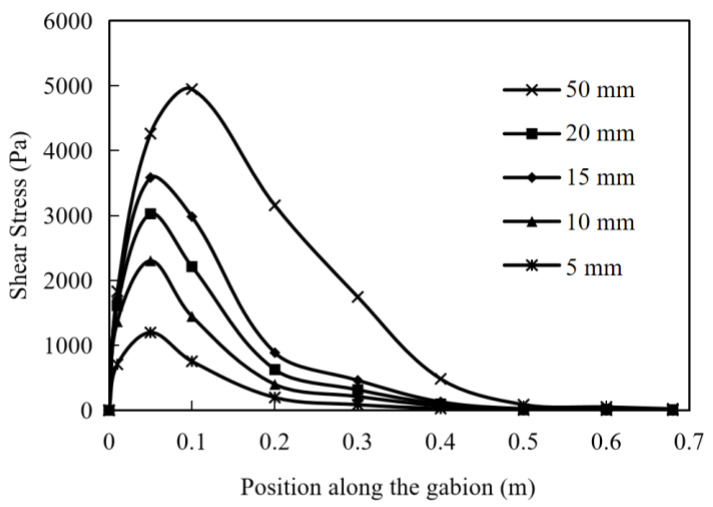
Shear stress of soil at pullout displacements of 5, 10, 15, 20 and 50 mm.

The shear stress of soil is an important indicator reflecting the friction performance of the interface. Under different pullout displacements, the shear stress distribution of the soil at the reinforcement layout is shown in Figure 12. Starting from the position of pulling force, the shear stress increases very fast and gradually decreases to 0 at a slower rate after reaching the peak value. As the pullout displacement gradually increases, the shear stress of the soil at various positions increases. In particular, it is evident that the horizontal position corresponding to the peak shear stress of the soil gradually shifts to the right when the pullout displacement reaches 50 mm. When the displacement is small, this characteristic is not significant. As the pullout displacement increases, the degree of pulling becomes deeper, and the reinforcement material mobilizes the more internal soil to resist through shear stress.

**Figure 13:**
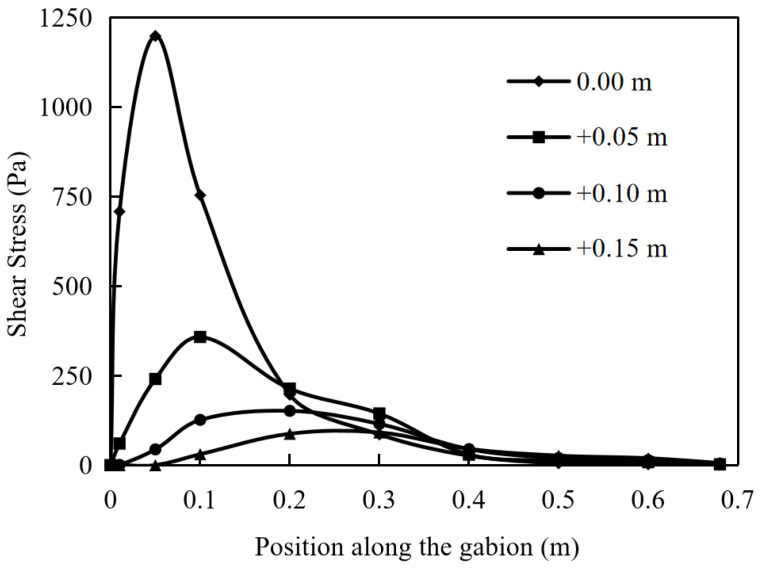
Shear stress of soil away from gabion of 0, 0.05, 0.10 and 0.15 m (pullout displacement: 5 mm).

**Figure 14:**
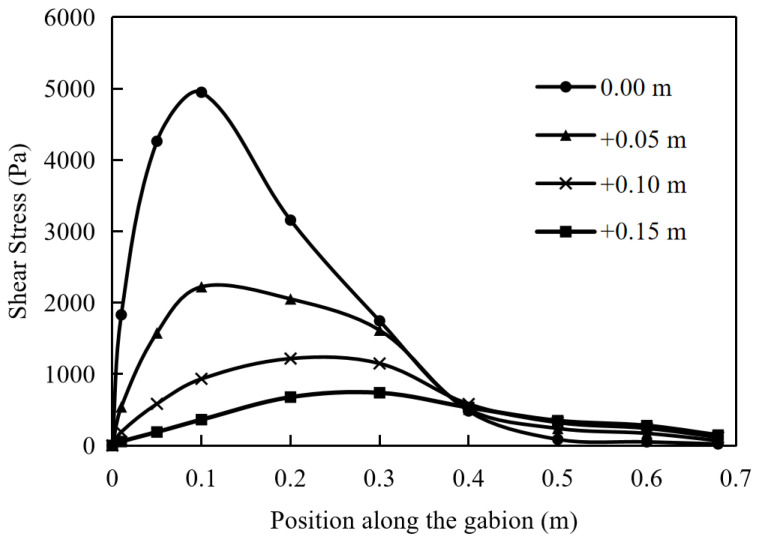
Shear stress of soil away from gabion of 0, 0.05, 0.10 and 0.15 m (pullout displacement: 50 mm).

When the pullout displacement is 5 mm and 50 mm, the shear stress distribution of the soil along the depth direction (away from the gabion) is shown in Figure 13 and Figure 14. The shear stress of the soil decreases non-linearly with the increase in the distance from the gabion. When the pullout displacement is 5 mm, the maximum shear stress of the soil at a distance of 0.15 m from the gabion is 7.6% of that at the arrangement. As the displacement is 50 mm, the maximum shear stress of the soil at a distance of 0.15 m from the gabion is 14.9% of that at the arrangement. When the shear stress of soil increases, its influence range in the vertical direction will be larger.

### 5.5. Pullout Curves at Different Rates

**Figure 15:**
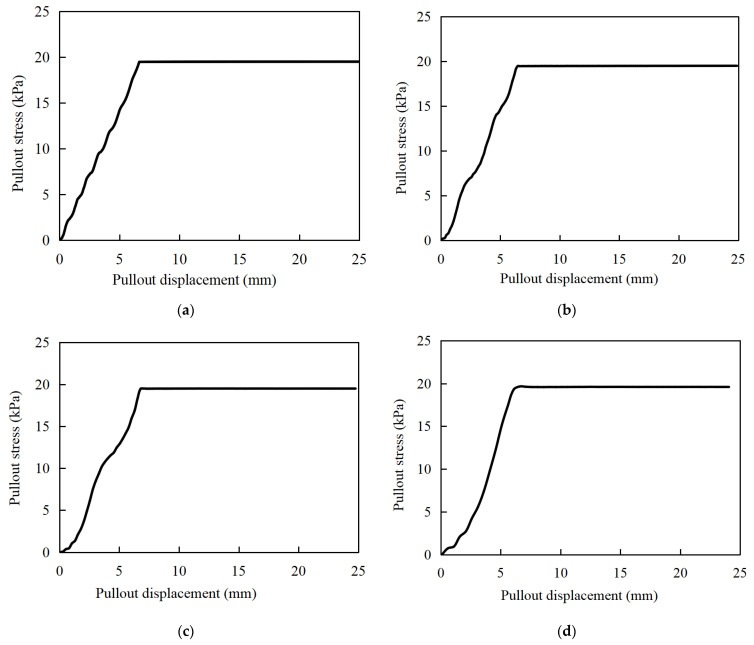
Pullout curves at different rates. (**a**) v = 0.01 mm/s; (**b**) v = 0.03 m/s; (**c**) v = 0.05 mm/s; (**d**) v = 0.10 mm/s.

In the above analysis, it is noticed that the pullout curves obtained from the numerical simulation are not smooth in the fast growth phase compared to the laboratory pullout tests. Due to the computational steps used by FLAC3D 6.0, the numerical results exhibit a stepped pattern of regular fluctuations. Numerical simulation tests were carried out at different pullout rates under the normal stress of 15 kPa. Pullout curves at different pulling rates are shown in Figure 15, which is a clear proof of this conjecture.

The critical pullout stress and corresponding critical displacement are almost not affected by different pulling rates. However, the calculation steps required to reach the critical displacement are different due to the different pullout rates. The smaller the drawing rate is, the more calculation steps are needed, the more stable differential cycles are obtained, and the more obvious is the overall pattern. As the pulling rate increases to 0.05 mm/s, only one complete differential cycle is obtained for the pulling curve in the fast growth phase, resulting in a distortion of the pullout curve. Further, the higher the normal stress in the pullout test is, the less compatible the pullout curve will be. Therefore, more attention should be paid to this point when performing the FLAC3D 6.0 simulation.

## 6. Conclusions

A pullout test of a double-twisted hexagonal gabion mesh with weathered coarse-grained red sandstone was carried out. Corresponding to the experimental model, a numerical model was constructed based on the FLAC3D 6.0 platform. Further, the relationships between the displacement and stress of gabion mesh and filler during the pulling process were analyzed, and the interaction mechanisms of gabion mesh and filler are explained.

The pullout curves can be divided into three stages: rapid growth stage, development transition stage and yielding stabilization stage. There is a mutual extrusion between each hexagonal mesh of the gabion and the surrounding soil under the normal stress, which increases the surface friction and the occlusion force generated by the interlocking action between soil particles.

The critical displacement corresponding to peak pullout stress increases with the increase in the normal stress. The normal stress determines the magnitude of the shear stress at the interface between the gabion mesh and the soil, and the displacement distribution of the gabion mesh is not significantly affected by the normal stress when the applied normal stress is within a range of 7–20 kPa.

## Figures and Tables

**Table 1:** Basic physics index of soil.

Properties	Value
Water content (%)	14.65
Relative density	2.74
Cohesion (kPa)	25
Internal friction angle (°)	21
Optimum water content (%)	18.13
Maximum dry density (g/cm^3^)	1.73

**Table 2:** The particle size distribution of weathered red sandstone soil.

The Content of Particle Size Fractions (mm)/%					Controlled Particle Size/mm			Gradation Coefficient	
<5	5~10	10~20	20~40	40~60	d_60_	d_30_	d_10_	C_u_	C_c_
14.2	10.3	15.3	20.2	18.6	40.08	13.16	3.08	13.02	1.40

**Table 3:** Technical indexes of gabion mesh.

Technical Indexes	Value
Mesh Size	8 mm × 10 mm
Length	680 mm
Diameter of gabion wire	2.2 mm
Diameter of edge wire	2.7 mm
Tensile test fracture strength	25.90 kN/m

**Table 4:** Gabion mesh frictional parameters.

Reinforced Material	Cohesion (kPa)	Interface Friction Angle (°)
Gabion mesh	10.9	25.3

**Table 5:** Comparison of peak pullout stress in laboratory and numerical simulation.

Normal Stress	7 kPa	10 kPa	15 kPa	20 kPa
Pullout test	14.10	15.90	17.90	20.40
Numerical simulation	15.65	17.35	19.70	22.70
Error	10.99%	9.12%	10.06%	11.27%

## Data Availability

The data presented in this study may be available on reasonable request from the corresponding author.
